# Patient-centered outcomes and outcome measurements for people aged 65 years and older—a scoping review

**DOI:** 10.1186/s12877-024-05134-7

**Published:** 2024-06-18

**Authors:** Åsa G. Andersson, Lisabet Dahlkvist, Lisa Kurland

**Affiliations:** 1 Department of Geriatrics, School of Medical Sciences, Faculty of Medicine and Health, Örebro University, Örebro, Sweden; 2 Centre of Clinical Research, Region Värmland, Karlstad, Sweden; 3 Department of Emergency, School of Medical Sciences, Faculty of Medicineand , Health Örebro University, Örebro, Sweden

**Keywords:** Older people, Patient-centered, Patient-centered outcomes, Patient-centered outcomes measurements, What matters the most

## Abstract

**Introduction:**

The aging population is a challenge for the healthcare system that must identify strategies that meet their needs. Practicing patient-centered care has been shown beneficial for this patient-group. The effect of patient-centered care is called patient-centered outcomes and can be appraised using outcomes measurements.

**Objectives:**

The main aim was to review and map existing knowledge related to patient-centered outcomes and patient-centered outcomes measurements for older people, as well as identify key-concepts and knowledge-gaps. The research questions were: How can patient-centered outcomes for older people be measured, and which patient-centered outcomes matters the most for the older people?

**Study design:**

Scoping review.

**Methods:**

Search for relevant publications in electronical databases, grey literature databases and websites from year 2000 to 2021. Two reviewers independently screened titles and abstracts, followed by full text review and extraction of data using a data extraction framework.

**Results:**

Eighteen studies were included, of which six with involvement of patients and/or experts in the process on determine the outcomes. Outcomes that matter the most to older people was interpreted as: access to- and experience of care, autonomy and control, cognition, daily living, emotional health, falls, general health, medications, overall survival, pain, participation in decision making, physical function, physical health, place of death, social role function, symptom burden, and time spent in hospital. The most frequently mentioned/used outcomes measurements tools were the Adult Social Care Outcomes Toolkit (ASCOT), EQ-5D, Gait Speed, Katz- ADL index, Patient Health Questionnaire (PHQ9), SF/RAND-36 and 4-Item Screening Zarit Burden Interview.

**Conclusions:**

Few studies have investigated the older people’s opinion of what matters the most to them, which forms a knowledge-gap in the field. Future research should focus on providing older people a stronger voice in what they think matters the most to them.

**Supplementary Information:**

The online version contains supplementary material available at 10.1186/s12877-024-05134-7.

## Introduction

Both the number and proportion of older people is increasing in most countries. In 2019, there were 703 million people aged 65 years and older in the world, corresponding to nine percent of the population, and estimates predict that this number will have doubled by 2050 [1]. An aging population is a challenge for the healthcare system [2], which was underscored by the coronavirus pandemic 2019 (COVID-19) [3]. Hence, the healthcare system needs to identify strategies to meet the needs of the growing proportion of older people in order to achieve a high quality of care [2], a central aspect of being the focus on patient-centered care and outcomes for older people [3].

The concept of patient-centered care was first introduced in the late 1980ies, and has come to gain impact on healthcare research [4]. The essence of patient-centered care can be captured by the question posed as “what matters to you?, rather than the more traditionally used, “what is the matter with you?”[5]. The patient-centered approach shifts the focus from clinical guidelines to the patient’s requests, experiences, and point of view – i.e. a shift of caring focus from healthcare-centered to patient-centered [5]. The effect of patient-centered care is patient-centered outcomes which can be measured by patient-centered outcome measurements [6]. There are to our knowledge no prior systematic reviews studying both patient-centered outcomes and patient-centered outcome measurements specific to older people.

Studies regarding patient-centered outcomes and patient-centered outcome measurements typically focus on a specific condition, disease, or event, such as stroke, bladder cancer, anemia or asthma [7–10]. Previous studies have focused on patient-centered outcomes and patient-centered outcome measurements in general [11–13], but few studies have focused on patient-centered outcomes and how to measure these for older people [14]. Old people often have complex needs [15] motivating a holistic, patient-centered approach [5]. Therefore, this review has focused on publications reflecting a general approach among unselected patient populations, i.e. not on specific conditions.

The aim of the current scoping review was to review and map the existing knowledge regarding patient-centered outcomes and patient-centered outcome measurements for people 65 years of age and above, representing an unselected patient population, as well as to identify key-concepts and knowledge-gaps.

## Methods

### Study design

The scooping review method was chosen as a form of knowledge synthesis to provide an overview of available knowledge in relation to the research questions: which patient-centered outcomes matter the most for older people? How can these patient-centered outcomes for older people be measured? [16].

### Protocol and registration

A review protocol was established in accordance with the framework proposed by Arksey and O’Malley [17], Levac et al. [18] and the Joanna Briggs Institute [16]. The review protocol was registered in April 2021 on the Open Science Framework (OSF) website [19].

### Eligibility criteria

An initial exploratory search of publications relevant to the topic was conducted prior to the registration of the review protocol, and the results discussed in the research group. Based on the exploratory search, the following eligibility criteria were defined:

- ▪ Main topic/core concept of the publication: Patient-centered outcomes and/or patient-centered outcome measurements.
- ▪ Study context: A broad context was chosen to limit the risk of overseeing relevant evidence anywhere in the health care system.
- ▪ Study population: People aged 65 years and older. The age-limit was chosen since the cutoff age for older people in research commonly is 65 years and older [20, 21]. An unselected study population, i.e. no specific medical condition, since the aim was to investigate the population of older people in general, and not in relation to a given condition. Publications using the term “multimorbidity”, which is common among the study population of interest [15], were included.
- ▪ Type of publication: Peer reviewed original articles and systematic reviews.
- ▪ Time frame: From year 2000 to 2021.
- ▪ Language: English.

Exclusion criteria:

▪ Conference abstracts, book reviews, commentaries, and editorial publications.

▪ Publications that focus on a specific disease or event.

### Search

The search strategy was developed and executed in collaboration with experienced librarians.

The full electronic search strategy for the database PubMed is shown in Appendix No 1. Grey literature was searched in databases and websites relevant to the topic. This was done in the same manner as for the electronic databases, however, the search strategy was adapted to the specific database or website and its search function.

### Information sources

In 2021 a search for previous reviews related to the topic of patient-centered outcomes and patient-centered outcome measurements for older people was conducted in the databases PubMed and Joanna Briggs Institute. The search generated five systematic reviews of patient-centered care and patient-centered outcomes for older people [22–26] and three scoping reviews regarding patient-centered outcomes, however, not specific to older people [27–29].

**Fig. 1:**
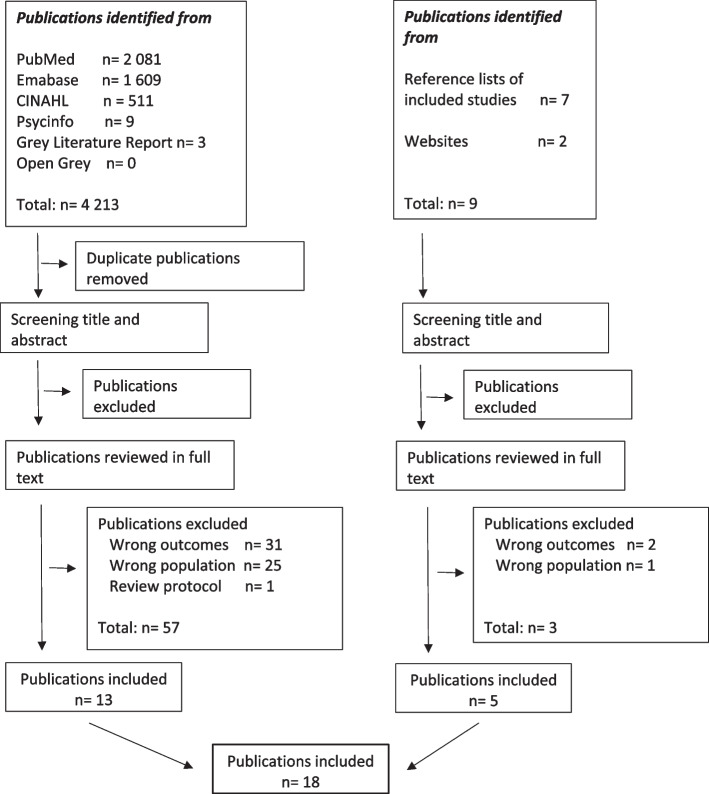
Selection of publications. Flowchart according to PRISMA [30]

Relevant publications were searched in 2021 using the following electronic databases: MEDLINE/PubMed, Cumulative Index to Nursing and Allied Health Literature (CINAHL), PsycINFO and EMBASE. Grey literature sources were searched in the databases Grey Literature Report and Open Grey and in the following websites: the “Patient-Centered Outcomes Research Institute” (pcori.org), the “Agency for Healthcare Research and Quality” (ahrq.gov) and “Patient Centered Outcomes Research” (pcor.org.uk). Removal of duplicates was performed by the librarian and the remaining publications were consolidated in the reference management software Covidence®. The reference lists of the initially included publications (*n* = 13) were hand searched to limit the risk of overlooking relevant publications. An additional five relevant publications were identified and included in Covidence (Fig. 1).

### Selection of publications

The review process consisted of two levels of screening. At first, two reviewers (LD, ÅA) independently screened publications for titles and abstracts in accordance with inclusion and exclusion criteria. Second, the publications of interest were read independently and reviewed in full text by the two reviewers, with application of the eligibility- and exclusion criteria. Disagreements regarding the eligibility of a publication were discussed and a third reviewer acted as an arbitrator (LK) when consensus was not reached.

### Data extraction

The data extraction framework was developed prior to the registration of the review protocol, Appendix No 2. A pilot test on one of the included publications was performed to ensure consistent application of the data extraction framework. No revision of the data extraction framework was needed after the pilot test. The two reviewers independently extracted the data from the included publications using the data extraction framework. The data was compared between the two reviewers. Any differences in extracted data were discussed (LD and ÅA), and a third reviewer (LK) was consulted if consensus was not reached.

### Data collection

Data was extracted in accordance with the data extraction framework, (Appendix No 2).

### Synthesis of results

Data was reviewed and synthesized to obtain an accessible overview and to answer the research aims. Information from the included publications was synthesized using a pragmatic narrative approach in the following steps. First, the publications were categorized based on country of origin, year of publication, study design and context in the health care system. Secondly, patient-centered outcomes and outcome measurement tools were categorized based on the core concept of the outcome of the publication into the following categories: access to and experience of care, carer needs, cognition, daily living, emotional health, physical health, quality of life and others. Thereafter, an additional consolidation of results to present the most common expressions of what matters the most to the older people and how this can be measured is presented. Finally, the involvement of the study population was categorized based on study participants as: participation of older people, older people and experts and experts.

## Results

### Selection of sources of publications

Eighteen publications of the total of 4 222 publications were included. The identification and selection of publications is presented in Fig. 1.

### Characteristics of included publications

Characteristics of the included studies are presented in Appendix No 3 and include the information according to the data extraction framework (Appendix No 2) with the exception of study outcomes.

### Results from included publications

Patient-centered outcomes, patient-centered outcome measurements and the involvement of the study population in the process of determining which outcomes to measure and which matters the most, are presented in Table 1.

**Table 1:** Results of included publications (*n* = 18)

**Title**	**Patient-centered outcomes**	**Patient-centered outcomes measurement tools**	**Study populations involvement in process**
Standard set of health outcome measures for older persons [14]	1) Tier 1: Overall survival, place of death, frailty 2) Tier 2: Polypharmacy, falls, participation in decision making, time spent in hospital 3) Tier 3: Loneliness and isolation, activities of daily living, pain, mood and emotional health, autonomy and control, carer burden	1) Tier 1: Administrative- and clinical data, Clinical Frailty Scale (CFS) 2) Tier 2: Administrative- and clinical data, patient reported 3) Tier 3: UCLA-3-item scale, SF-36, Gait speed, Adult Social Care Outcomes Toolkit, 4-Item screening Zarit Burden Interview	Experts and patient-representatives involved in the decision on which outcomes to use and which matters the most
Association between Continuity of Care and Health-Related Quality of Life [31]	1)Self-reported health-status 2)Physical function 3)Emotional well-being	36-item Rand questionnare	Study-population not involved in the decision on which outcomes to use and which matters the most
Association of patient-centered outcomes with patient-reported and ICD-9 based morbidity measures [32]	1) Self-reported health status 2) Physical and mental well-being 3) Feeling overwhelmed by one’s medical conditions 4) Experiencing financial constraints caused by health care costs 5) Level of general self-efficacy	1) Self-reported health status and physical and mental well-being measured by: RAND 36 2) Remaining measured by: instrument developed by the authors	Study-population not involved in the decision on which outcomes to use and which matters the most
Personal Functional Goals: A New Approach to Assessing Patient-Centered Outcomes [33]	1) Physical and mental health: Energy, strength, appearance, pain, mood, coping with stress, other illnesses etc 2) Daily living: Household chores, bathe self, walk unassisted, visit with family and friends etc 3) Other: Stay active, live longer, feel better, live a more fulfilling life etc	Personal Functional Goals Interview	Study-population involved in the decision on which outcomes to use and which matters the most
What Matters, and What Matters Most, for Change in Life Satisfaction in the Oldest-Old? A Study over 6 Years among Individuals 80 + [34]	Life satisfaction	The Life Satisfaction Index-Z (LSI-Z)	Study-population not involved in the decision on which outcomes to use and which matters the most
Measured outcomes of chronic care programs for older adults: a systematic review [35]	1) Self-reported health 2) Well-being 3) Cognitive function/physical function/ADL/IADL function, incontinence 4) Health related quality of life, physical quality of life 5) Depression/anxiety 6) Relationships/social functioning 7) Care needs/complexity of care needs 8) Frailty 9) Caregiver burden/strain/depression/quality of life/ self-rated burden of care etc	1) Activities of Daily Living (ADL) and Instrumental Activities of Daily Living (IADL) measured by: Groningen Activity Restriction Scale, Katz ADL index, Avlund scale, Shuttle-walk test, chair stand test, 2.45 m up and go, Physical function survey 2) Frailty measured by: Groningen Frailty Index (GFI) self-report version 3) Psychological wellbeing measured by: Groningen Well-being Indicator (GWI) 4) Health-related quality of life measured by: EQ-5D, SF-12, RAND-36, SF-36, 24 item HRQL from SF-36 and QUAL-E 5) No specification regarding how remaining outcomes were measured	Study-population not involved in the decision on which outcomes to use and which matters the most
Effects of a continuum of care intervention on frail older persons’ life satisfaction: a randomized controlled study [36]	1) Life satisfaction 2) Illness 3) Functional ability 4) Health 5) Medication 6) Quality of care 7) Frailty	1) Life satisfaction measured by: Life satisfaction 11 (LiSat-11) 2) Remaining outcomes not specified how they were measured	Study-population not involved in the decision on which outcomes to use and which matters the most
The impact of patient knowledge of patient-centered medication label content on quality of life among older adults [37]	Quality of life	EQ-5D, EQ-VAS	Study-population not involved in the decision on which outcomes to use and which matters the most
Resident Outcomes in Small-House Nursing homes: A Longitudinal Evaluation of the Initial Green House program [38]	1) Quality of life: Physical comfort, functional competence, privacy, dignity, meaningful activity, relationship, autonomy, food enjoyment, spiritual well-being, security, individuality 2) Health and functioning: Rating of health, activities of daily living and instrumental activities of daily living 3) Satisfaction: Satisfaction with nursing home as a place to live and a place to receive care 4) Emotional well-being 5) Social activity 6) Quality of care	1) Quality of care measured by: Indicators derived from Minimum Data Set assessments 2) Remaining outcomes measured by: Questions and scales developed by the authors	Study-population not involved in the decision on which outcomes to use and which matters the most
First insights on value-based healthcare of elders using ICHOM older person standard set reporting [39]	From ICHOM Older Person Standard set: 1) Tier 1: Overall survival, place of death, frailty 2) Tier 2: Polypharmacy, falls, participation in decision making, time spent in hospital 3) Tier 3: Loneliness and isolation, activities of daily living, pain, mood and emotional health, autonomy and control, carer burden Cognitive function Comorbidity	From ICHOM Older Person Standard set: 1) Tier 1: Administrative- and clinical data, CFS 2) Tier 2: Administrative- and clinical data, patient reported 3) Tier 3: UCLA-3-item scale, SF-36, Gait speed, Adult Social Care Outcomes Toolkit, 4-Item screening Zarit Burden Interview Cognitive function measured with The Montreal Cognitive Assessment (MoCA) Comorbidity measured with Charlson Comorbidity Index	Study-population not involved in the decision on which outcomes to use and which matters the most
Home Time as a Patient-Centered Outcome in Administrative Claims Data [40]	1) Self-rated health 2) Mobility 3) Depression 4) Social activity 5) Self-care	N/A	Study-population not involved in the decision on which outcomes to use and which matters the most
A Home-Based Care Research Agenda by and for Homebound Older Adults and Caregivers [41]	1) Out-of-pocket costs of caregiving 2) Access to home-based care and related policy issues 3) Relationships with doctors 4) Getting to know patients and caregivers as individuals 5) Understanding patients and caregiver needs and well-being 6) Specialist care in the home 7) Challenges receiving care outside the come 8) Communication 9) Issues regarding paid caregivers 10) Home as a therapeutic place 11) Quality of nursing homes 12) Technology in the home 13) Dementia 14) Delivery services	N/A	Patients and careers involved in the decision on which outcomes to use and which matters the most
Important care and activity preferences in a nationally representative sample of nursing home residents [42]	1) Care- and activity preferences 2) Function 3) Depression 4) Cognitive impairment	1) Care- and activity preferences measured by: Preferences Assesment Tool (PAT) from Minimum Data Set 3.0 (MDS) 2) Activity measured by: MDS ADL-Long form summary score 3) Depression measured by: Patient Health Questionnaire (PHQ9) 4) Cognitive impairment measured by: Brief Interview for Mental Status (BIMS)	Study-population not involved in the decision on which outcomes to use and which matters the most
Universal health outcome measures for older persons with multiple chronic conditions [43]	1) General health 2) Pain 3) Fatigue 4) Physical and mental health 5) Social role function 6) Gait speed 7) Symptom burden 8) Depression 9) Anxiety 10) Daily activities	1) General health measured by: Short-form 8 (SF-8), 36 (SF-36), the Patient Reported Outcomes Measurement Information System29-item Health Profile (PROMIS-29) 2) Physical health measured by: Condensed Memorial Symptom Assessment Scale (CMAS) 3) Physical function and mobility measured by: Gait speed, PROMIS physical function with mobility aid short form, Activities of Daily Living (ADL) and Instrumental Activities of Daily Living (IADL) questionaries 4) Mental health measured by: SF-36, Patient Health Questionnaire (PHQ-9), Generalized Anxiety Disorder 7 (GAD-7)	Experts involved in the decision on which outcomes to use
Toward Patient-Centered Care: A Systematic Review of Older Adults’ Views of Quality Emergency Care [44]	1) Role of health care providers 2) Communication and patient education 3) Barriers to communication 4) Wait times 5) Physical needs in the emergency care setting 6) General elder care needs 7) Care transitions	N/A	Study-population involved in the decision on which outcomes to use and which matters the most
Developing a Senior Healthcare Practice Using the Chronic Care Model. Effect on Physical Function and Health related Quality of life [45]	1) Physical function 2) Health-related quality of life	1) Physical function measured by: Physical function survey 2) Health-related quality of life measured by: 24- Item health-related quality of life (derived from the 36-Item medical outcomes study short form health survey database)	Study-population not involved in the decision on which outcomes to use and which matters the most
Effects of case management in community aged care on client and carer outcomes: a systematic review of randomized trials and comparative observational studies [46]	Client/patient outcomes: 1) Mortality 2) Physical/cognitive functioning 3) Medical conditions 4) Behavioral problems 5) Unmet service needs 6) Physiological health/well-being 7) Satisfaction with care Carer outcomes: 1) Stress/burden 2) Satisfaction with care 3) Psychological health/well-being 4) Social consequences	1) Functional status measured by: Katz ADL, Lawton and Brody´s IADL scale 2) Psychiatric symptoms and behavioral disturbance measured by: Neuropsychiatric Inventory (NPI) 3) Well-being measured by: Personal well-being Index-Intellectual Disability (PWI-ID) 4) Depression measured by: Short form Geriatric Depression scale, Cornell Scale for Depression in Dementia (CSDD) 5) Cognitive functioning measured by: Mini Mental State Examination (MMSE) 6) Carer burden measured by: Zarit Carer Burden Interview 7) Carer psychological health measured by: General health questionnare (GHQ) 8) Carer subjective quality of life measured by: Personal well-being Index for Adults (PWI-As) 9) Client and carer health status measured by SF-36	Study-population not involved in the decision on which outcomes to use and which matters the most
A patient-centered research agenda for the care of the acutely ill older patient [47]	1) Advanced care planning 2) Care Transitions 3) Delirium 4) Dementia 5) Depression 6) Medications 7) Models of Care 8) Physical function 9) Surgery 10) Training	N/A	Patient- and caregivers’ representatives involved in the decision on which outcomes to use and which matters the most

### Synthesis of results

#### Publication information

Over half of the publications were written in the USA, published after year 2010 and the most common context was the community setting. Several different study designs/methods were used. Information regarding the publications is summarized in Table 2.

**Table 2:** Publication information

Country	Year of publication	Study design/method	Context
Australia (*n* = 1)	2000–2010 (*n* = 4)	Prospective studies (*n* = 7)	Community and healthcare (*n* = 16)
Canada (*n* = 1)	2011–2021 (*n* = 14)	Retrospective studies (*n* = 2)	Emergency care/medicine (*n* = 2)
China/USA (*n* = 1)		Randomized control study (*n* = 1)	
Sweden (*n* = 1)		Qualitative studies (*n* = 3)	
Sweden/USA (*n* = 1)		Systematic reviews (*n* = 3)	
Taiwan (*n* = 1)		Consensus meeting (*n* = 1)	
UK (*n* = 1)		Modified Delphi Technique (*n* = 1)	
USA (*n* = 11)			

### Synthesis of patient-centered outcomes and outcome measurement tools

The following patient-centered outcomes were the most frequently mentioned: access to care, activities of daily living (ADL), care needs, carer burden, cognitive function, communication, depression, emotional well-being, health, instrumental activities of daily living (IADL), medications, physical function, quality of care, quality of life and social activity. The most frequently mentioned measurement tools were the Adult Social Care Outcomes Toolkit (ASCOT), EQ-5D, Gait Speed, Katz- ADL index, Patient Health Questionnaire (PHQ9), SF/RAND-36 and 4-Item Screening Zarit Burden Interview.

Tables 3 and 4 present a synthesis of patient centered outcomes and measurement tools. The main categories were access to- and experience of care, carer needs, cognition, daily living, emotional health, physical health, and quality of life.

**Table 3:** Patient-centered outcomes

Assess to- and experience of care	Carer needs	Cognition	Daily living	Emotional health	Physical health	Quality of life	Others
Care needs (*n* = 6)	Carer burden (*n* = 11)	Cognitive function (*n* = 7)	ADL/IADL (*n* = 10)	Emotional well-being (*n* = 8)	Health (*n* = 9)	Quality of life (*n* = 7)	Medi-cations (*n* = 5)
Quality of care (*n* = 5)			Physical function (*n *= 6)	Depression (*n* = 5)	Frailty (*n* = 4)		Place of death (*n* = 2)
Access to care (*n* = 4)			Autonomy and control (*n* = 4)	Anxiety (*n* = 2)	Mortality (*n* = 3)		Symptom burden
Communication (*n* = 4)			Social activity (*n* = 4)	Loneliness and isolation (*n* = 2)	Pain (*n* = 3)		
Care transitions (*n* = 2)			Self-care	Behavioral problems	Physical health (*n* = 3)		
Costs (*n* = 2)			Home as a therapeutic place		Comorbidity (*n* = 2)		
Time spent in hospital (*n* = 2)			Relationships		Falls (*n* = 2)		
Care planning			Technology in the home		Fatigue		
Delivery services			Training		Incontinence		
Getting to know patients and caregivers as individuals					Surgery		
Issues regarding paid caregivers							
Physical needs in the emergency care							
Relationships with doctors							
Role of health care providers							
Wait times							

*(*n* =) = number of times the patient-centered outcomes were mentioned/used in the reviewed studies

**Table 4:** Patient-centered outcomes measurement tools

Assess to- and experience of care	Carer needs	Cognition	Daily living	Emotional health	Physical health	Quality of life	Others
Preferences Assesment Tool (PAT) from MDS	Zarit Carer Burden Interview (*n* = 3)	Brief Interview for Mental Status (BIMS)	Gait speed (*n* = 3)	Patient Health Questionnaire (PHQ9) (*n* = 2)	Charlson Comorbidity Index	Short form 36 (SF-36)/RAND-36 (*n* = 7)	Personal functional goals interview
Minimum Data Set assessments 3.0 (MDS)		Mini Mental State Examination (MMSE)	Katz ADL index (*n* = 2)	UCLA-3-item loneliness scale (*n* = 2)	Clinical Frailty Scale (CFS)	Adult Social Care Outcomes toolkit (ASCOT) (*n* = 2)	
		Montreal Cognitive Assessment (MoCA)	ADL and IADL questionaries	Generalized Anxiety Disorder 7 (GAD-7)	Condensed Memorial Symptom Assessment Scale (CMAS)	EQ-5D (*n* = 2)	
			ADL-Long form summary score	Groningen Well-being Indicator (GWI)	Groningen Frailty Index (GFI) self-report version	EQ-VAS	
			Avlund scale		Physical function survey	Life Satisfaction Index -Z (LSI-Z)	
			Chair stand test			LiSat-11	
			Groningen Activity Restriction Scale			Patient Reported Outcomes Measurement Information System29-item Health Profile (PROMIS-29)	
			Lawton and Brody´s IADL scale			QUAL-E	
			Shuttle-walk test 2.45 m up and go			Short-form 8 (SF-8)	
						Short form 12 (SF-12)	
						24-Item health-related quality of life	

*(*n* =) = number of times the patient-centered outcomes were mentioned/used in the reviewed studies

### Study population and what matters the most

**Fig. 2:**
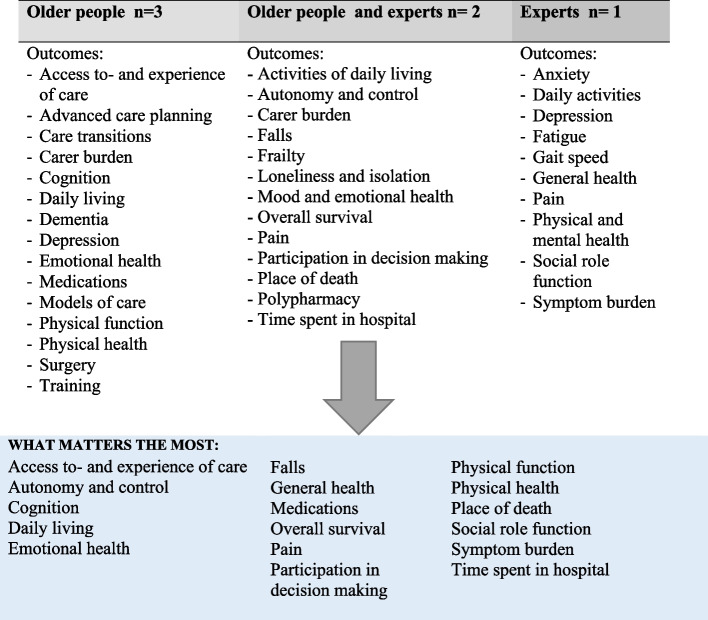
Patient-centered outcomes that matter the most to older people

Older people were not involved in the process of determining which outcomes mattered the most and how to measure them in 12 of the 18 included publications. Three of the studies involved older people as participants, one used experts in the field as patient representatives, and two involved both older people and experts. The synthesis of the results showed that the outcomes that matter most to older people were: access to- and experience of care, autonomy and control, cognition, daily living, emotional health, falls, general health, medications, overall survival, pain, participation in decision making, physical function, physical health, place of death, social role function, symptom burden and time spent in hospital, (Fig. 2).

## Discussion

The current scoping review aimed to explore the knowledge in the field of patient-centered outcomes and measurements for older people. The results showed that the outcomes that matter the most to older people were: access to- and experience of care; autonomy and control; cognition; daily living; emotional health; falls; general health; medications; overall survival; pain; participation in decision making; physical function; physical health; place of death; social role function; symptom burden; and time spent in hospital. The Adult Social Care Outcomes Toolkit (ASCOT), EQ-5D, Gait Speed, Katz- ADL Index, Patient Health Questionnaire (PHQ9), SF/RAND-36 and 4-Item Screening Zarit Burden Interview were the measurement tools most frequently used to measure patient-centered outcomes for older people.

The patient-centered outcomes in the current review were consolidated into the main categories: access to- and experience of care, carer needs, cognition, daily living, emotional health, others, physical health, and quality of life. Researchers at the Picker Institute have described that patient-centered care is based on the following dimensions: respect for the patient´s values, preferences and expressed needs, information and education, access to care, emotional support to relieve fear and anxiety, involvement of family and friends, continuity and secure transitions between healthcare settings, physical comfort, and coordination of care [48]. Further, a NEJM Catalyst article has suggested the following dimensions; mission and values aligned with patient goals; care is collaborative, coordinated and accessible; physical comfort and emotional well-being are top priorities; patient and family viewpoint respected and valued; patient and family always included in decisions; family welcome in care setting; full transparency and fast delivery of information [49]. The dimensions of patient centered care as suggested by the Picker Institute and the NEJM Catalyst article capture the same dimensions and support the current results. However, the current review identifies an important knowledge gap, i.e. that there are few studies actually including the target population: the older people themselves, while experts tend to speak on their behalf. Therefore, the list of what matters the most to older people, as presented here, should be considered as indicative. Future studies should involve older people to be able to answer to the question of what matters most to this population.

Attending to an ageing population is and will continue to be a challenge for the healthcare system [2]. Our results present how patient-centered outcomes can be measured and indicate that personal domains such as daily living and quality of life seem to be linked with the patient’s experienced health and well-being. Patient-centered care has been shown to lower the need of high-level emergency care and the risk of mortality for older people with multimorbidity [5] as well as reducing healthcare costs in multiple settings [50–52]. Including older people in the design of health care organization and caring pathways is needed in addition to including older people in scientific studies.

### Limitations

A strength of the study is the extensive literature search. A structured review has been carried out by two independent reviewers. A third reviewer was consulted if consensus was not reached. The major limitation is the inherent risk of limiting the literature search with the risk of not including relevant publications. An additional limitation is that the search strategy was not peer-reviewed. However, the search strategy was, in addition to the research team, developed in collaboration with experienced clinical librarians.

An additional limitation was the method used in the four steps of consolidation and results synthesis. A rigid method to analyze the level of evidence and further analyze the results was not applicable due to the limited number of publications which were included in the current study. Hence, a pragmatic, narrative approach was used.

Moreover, the search term “elderly” may be questioned, as the term old people has evolved to be the recommended terminology for the patient population of interest. However, this is a more recent development and we believe the results of the current study to be of interest despite this evolution.

## Conclusions

Patient-centered outcomes for older people can be summarized in the categories: access to- and experience of care; carer needs; cognition; daily living; emotional health; others; physical health; and quality of life. Patient-centered outcomes can be measured using several different measurement tools. Outcomes that matter the most to older people were: access to- and experience of care, autonomy and control, cognition, daily living, emotional health, falls, general health, medications, overall survival, pain, participation in decision making, physical function, physical health, place of death, social role function, symptom burden, and time spent in hospital. Importantly, few studies included the older people as the study population, despite patient centered aims. Future research should focus on providing the older people with a stronger voice in what they think matters the most to them.

### Supplementary Information

Supplementary Material 1.

## Data Availability

Inquiries for data access should be sent to the corresponding author, asa.andersson@oru.se, who will then contact the ethics board at Örebro University for permission to openly share the data.

## Abbreviations

ADL: Activities of daily life
ASCOT: Adult Social Care Outcomes Toolkit
BIMS: Brief Interview for Mental Status
CFS: Clinical Frailty Scale
CMAS: Condensed Memorial Symptom Assessment Scale
EQ-5D: EuroQol 5 dimensions
GAD-7: Generalized Anxiety Disorder 7
GFI: Groningen Frailty Index
GWI: Groningen Well-being Indicator
IADL: Instrumental activities of daily living
LiSat-11: Life Satisfaction Questionnaire
LSI: Life Satisfaction Index -Z (LSI-Z)
MDS: Minimum Data Set assessments
MMSE: Mini Mental State Examination
MoCA: Montreal Cognitive Assessment
PAT: Preferences Assesment Tool
PHQ-9: Patient Health Questionnaire
PROMIS-29: Patient Reported Outcomes Measurement Information System 29-item Health Profile
QUAL-E: Quality of life at the end of life
SF/RAND-36: 36 Item Short Form Survey
