# Supplementary material for: Patient-centered outcomes and outcome measurements for people aged 65 years and older—a scoping review
Source: BMC Geriatr. 2024 Jun 18;24:528. doi: 10.1186/s12877-024-05134-7 (PMC11186133; doi:10.1186/s12877-024-05134-7)
Supplement: Supplementary file 1 — Supplementary Material 1. [file 12877_2024_5134_MOESM1_ESM.docx]

**Appendix**

**No 1.** Search Strategy for PubMed 2021 12 14

| Search terms | | | Number of hits |
| --- | --- | --- | --- |
| Outcome Measurments | | | |
|  | 1 | (Outcome Assessment, Health Care [MeSH Terms]) OR (Patient Reported Outcome Measures [MeSH Terms]) | 1,251,991 |
|  | 2 | "Outcomes Assessment*"[Title/Abstract] OR "Outcome Assessment*"[Title/Abstract] OR "Outcome Research"[Title/Abstract] OR "Outcomes Research"[Title/Abstract] OR "Outcome Stud*"[Title/Abstract] OR "Outcome Measure*"[Title/Abstract] OR PROM[Title/Abstract] OR "Patient Reported Outcome*"[Title/Abstract] OR "Patient Reported Outcome Measure*"[Title/Abstract] OR "Patient-Reported Outcome*"[Title/Abstract] | 296,148 |
|  | 3 | 1 OR 2 | 1,467,294 |
| Patient-centered | | | |
|  | 4 | "What matters the most"[Title/Abstract] OR "patient-centered"[Title/Abstract] OR "Patient centered"[Title/Abstract] OR "value-based healthcare"[Title/Abstract] OR "health-care system"[Title/Abstract] | 54,612 |
| Elderly | | | |
|  | 5 | ((Aged [MeSH Terms]) OR (Aged, 65 and over [MeSH Terms])) OR (Frail Elderly [MeSH Terms]) | 3,347,005 |
|  | 6 | Old [Title/Abstract] OR Elderly [Title/Abstract] OR Aged [Title/Abstract] | 1,913,428 |
|  | 7 | 5 OR 6 | 4,728,993 |
| Combined sets | | | |
|  | 8 | 3 AND 4 AND 7 | 2,218 |
| Limits: Time 2000-, English language | | | |
|  | 9 | 3 AND 4 AND 7 | 2,081 |

**No 2. Data extraction framework**

| Main category | | |
| --- | --- | --- |
| 1 | Authors | |
| 2 | Date of publication | |
| 3 | Title | |
| 4 | Journal | |
| 5 | Country | |
| 6 | Objective(s) | |
| 7 | Study design | |
| 8 | Methodology | |
| 9 | Context | |
| 10 | Study population and sample size | |
| 11 | Data collection methods | |
| 12 | Data analysis | |
| 13 | Conclusion | |
| 14 | Outcomes | |
|  | a. | Description and definition of patient-centered outcomes |
|  | b. | Description of patient-centered outcome measurements |
| 15 | Study population’s involvement in process | |

| **No 3.** Characteristics of included publications (n=18) | | | | | | | | | |
| --- | --- | --- | --- | --- | --- | --- | --- | --- | --- |
| **Authors** | **Title** | **Publication date** | **Journal** | **Country** | **Obective(s)** | **Study design/**  **Methodology/**  **Data collection/**  **Analysis** | **Context** | **Study population** | **Conclusion** |
| Akpan *et al [1]* | Standard set of health outcome measures for older persons | 2018 | BMC Geriatrics | UK | Define a minimum set of outcomes for evaluating healthcare for older people | Modified Delphi Technique/  Literature reviews, surveys, focus groups, panel meetings | N/A | Working group with patient representatives, measurement experts, clinical, social, and psychological researchers (n= 31), focus group with members aged 68-89 years (n= 6) | The working group defined a standard set of recommended outcome measures that matter to older people |
| Bayliss *et al [2]* | Association between Continuity of Care and Health-Related Quality of Life | March/April  2017 | J Am Board Fam Med | USA | Investigate the longitudinal association between continuity of care and 3 domains of health-related quality of life | Prospective cohort study/ Phone surveys/ Mixed effects model, Multiple imputation | Health care delivery system | Patients aged $\geq$65 years with at least 1 year of enrollment before and at least 2 years of enrollment following the start; and had 3 of 10 common chronic medical conditions (n= 2 078, 961 completed the initial survey and 806 completed the follow-up) | Given the measurement burden of quality assessment, negative associations between potential quality metrics and care processes are informative. Systematic assessment of patient reported outcomes (PRO) can inform  patient centered multiple chronic medical conditions care. However, PRO scores should be used with caution as quality measures |

| Bayliss *et al [3]* | Association of patient-centered outcomes with patient-reported and ICD-9 based morbidity measures | March/April  2012 | Annals Of Family Medicine | USA. | Explore the independent contributions of self-reported morbidity and a morbidity measure based on administra-  tive diagnoses  for 2 types of health outcomes | Prospective cross-sectional cohort study/ Telephone surveys/Bivariate correlations, Multivariate analyses, Multivariate linear regression, Logistic regression, Negative binomial model | Health maintenance organization | Members of a health-maintenance organization aged $\geq$65 years, enrolled for at least 1 year, and had 3 or more of a list of 10 common chronic medical conditions  (n= 961) | A comprehensive assessment of morbidity requires both subjective and objective measurement of disease burden as well as an assessment of emotional symptoms. Such multidimensional morbidity measurement is particularly relevant for research or quality assessments involving the delivery of patient-centered care to complex patient populations. |
| --- | --- | --- | --- | --- | --- | --- | --- | --- | --- |

| Bearon *et al [4]* | Personal Functional Goals: A New Approach to Assessing Patient-Centered Outcomes | Septe-mber  2000 | The Journal of Applied Gerontol-ogy | USA | Identify personal functional goals for older adults in an exercise program | Qualitative study/ Three exploratory studies resulting in a list of 45 functional goals that were later tested/ Survey, interviews/ Reliability-test | Community | First exploratory study: Men and women aged $\geq$ 65 years (n= 47).  Second exploratory study: Men aged $\geq$ 69 years (n= 19).  Third exploratory study: Men aged $\geq$ 70 years (n= 159).  Test of Personal Functional Goals Interview Protocol: Men and women aged $\geq$65 years, who were participants in three exercise interventions studies (n= 39 (6 dropouts)) | A Personal Functional Goals Interview Protocol was developed |
| --- | --- | --- | --- | --- | --- | --- | --- | --- | --- |

| Berg *et al [5]* | What Matters, and What Matters Most, for Change in Life Satisfaction in the Oldest-Old? A Study over 6 Years among Individuals 80+ | March 2009 | Aging and Mental Health | USA, Sweden | Investigate both the unique contribution and the relative importance of a comprehensive set of variables for changes in life satisfaction among individuals in late life | Longitudinal cohort study/ Data from the Origins of Variance in the Old- Old (OCTO-Twin-study) of individuals aged $\geq$ 80 years/ Mixed, or multilevel, modeling (MLM) framework, Unconditional means model, Collinearity test | N/A | Participants were pair of twins aged >80 years (n= 412) | The results from the study question the notion of a life-long stability of life satisfaction |
| --- | --- | --- | --- | --- | --- | --- | --- | --- | --- |
| Drouin *et al [6]* | Measured outcomes of chronic care programs for older adults: a systematic review | October2015 | BMC Geriatrics | Canada | Determine to which extent published Chronic Care Model (CCM)/  Expanded Chronic Care Model (ECCM) initiatives were evaluated based on population, community, system, and individual-level outcomes | Systematic review/ Abstract and full text review/ Database search | Community and healthcare | All studies focused on people with an average age $\geq$ 65 years (n= 14) | Future efforts to test E/CCM interventions with seniors would be aided by more consistent outcome measures, greater attention to outcomes for the caregivers of older persons with chronic illness, and a greater focus on population and community impacts |

| Berglund *et al [7]* | Effects of a continuum of care intervention on frail older persons’ life satisfaction: a randomized controlled study | August  2014 | Journal of Clinical Nursing | Sweden | Analyze effects of a comprehensive  continuum of care on frail older persons’ life satisfaction, as compared to those receiving usual care | Randomised controll study/Geriatric assessment, case management etc. /  Face-to-face inter-  views using the LiSat-11 scale/ Power analysis, Chi-square tests, Mann-Whitney U-test | Municipal care, primary care, and hospital care | People living in their own homes, who visited the emergency department, aged $\geq$80 years, or aged 65-79 with a need for assistance in at least one activity of daily living and a minimum of one chronic illness  (n= 161) | A comprehensive continuum of care intervention comprising several components had a positive effect on frail older persons’ satisfaction with functional capacity, psychological health, and financial situation |
| --- | --- | --- | --- | --- | --- | --- | --- | --- | --- |

| Hong *et al [8]* | The impact of patient knowledge of patient-centered medication label content on quality of life among older adults | 2013 | Research in Social and Administ-rative Pharmacy | China, USA | To determine the extent of patient knowledge concerning patient-centered contents of medication labels (PCCMLs) and whether level of patient knowledge is associated with health outcomes | Prospective cross-sectional study/ Surveys/ Descriptive statistics, Analysis of variance, Correlation analysis Linear regression | Senior centers | People aged $\geq$65 years who were taking at least 1 medication  (n= 287) | Patient knowledge of medication use was associated with quality of life among older adults. New standardized labels should be designed to improve patient knowledge of PCCML information |
| --- | --- | --- | --- | --- | --- | --- | --- | --- | --- |

| Kane *et al [9]* | Resident Outcomes in Small-House Nursing homes: A Longitudinal Evaluation of the Initial Green House program | June  2007 | J Am Geriatric Soc | USA | To determine the effect of a small-house nursing home model, the Green House, on resident’s reported outcomes and quality of care | Longitudinal quasi-experimental study/ Green House residents were compared with a two comparison sites/ Interviews / Multivariate panel regression analyses using the random-effects regression models, Random-effects Tobit Model, Random-effects Ordered Probit regression models, Random-effects population-averaged linear models, Postestimation Wald test, Random-effects logit regression | Nursing homes | Residents from three different nursing homes, mean age >81 years (n= 120) | The Green House is a promising model to improve quality of life for nursing home residents, with implications for staff development and medical director roles |
| --- | --- | --- | --- | --- | --- | --- | --- | --- | --- |
| Lee *et al* [10] | First insights on value-based healthcare of elders using ICHOM older person standard  set reporting | 2020 | BMC Geriatrics | Taiwan | Report value-based health metrics in Taiwan using the ICHOM older person toolset | Prospective cross-sectional cohort study  / Student t test, Chi-square analysis, Multivariable logistic regression analyses | Community | Adults aged ≥ 65 years and with ≥ 3 chronic medical conditions  (n= 299) | The ICHOM Standard Set Older Person health outcome measures provide an opportunity to shift from a disease-centric medical paradigm to whole person-focused goals. Advanced age, chronic disease burden and cognitive impairment is important barriers to achieving high value-based healthcare status. |

| Lee *et al [11]* | Home Time as a Patient-Centered Outcome in Administrative Claims Data | Februa-ry 2019 | J Am Geriatric Soc | USA | Compare home time against existing patient-centered outcome measures | Retrospective cohort study/ Data from the Medicare Current Beneficiary Survey (MCBS)/ Kruskal-Wallis’s test, Chi-square test | Community | Medicare beneficiaries aged $\geq$65 years  (n= 4,594) | A loss in home time is associated with decline in several patient-centered outcome measures in community-dwelling Medicare beneficiaries |
| --- | --- | --- | --- | --- | --- | --- | --- | --- | --- |
| Leff *et al [12]* | A Home-Based Care Research Agenda by and for Homebound Older Adults and Caregivers | Februa-ry 2021 | Journal of Applied Gerontol-ogy | USA | Engage homebound older adults and their caregivers to develop a patient- and caregiver-centered research agenda relevant to their needs and perspectives | Qualitative study/ Survey, video conferences | Homebound elderly | Stakeholder advisory boards (SAB) members with patients aged  $\geq$ 66 years (n= 4) and  caregivers aged $\geq$ 58 years (n= 4) | The SAB members developed a patient- and caregiver centered  research agenda consisting of 14 research domains and 127 associated research questions. Domains related to access, cost, and aspects of communication were highly prioritized |

| Roberts *et al [13]* | Important care and activity preferences in a nationally representative sample of nursing home residents | January2018 | J Am Med Dir Assoc | USA. | Use data from the Minimum data set 3.0 (MDS) Preferences Assesment Tool (PAT) to describe: 1) overall resident preferences, 2) variation in preferences across items, and 3) variation in preferences across residents | Retrospective cohort study/ Data from admission Minimum Data Set 3.0 assessments/ Descriptive statistics, Proportional differences tests, Logistic regression | Nursing homes | Residents aged $\geq$65 years (n= 244,718) | PAT captures variation in preferences across items and residents |
| --- | --- | --- | --- | --- | --- | --- | --- | --- | --- |
| Salive *et al [14]* | Universal health outcome measures for older persons with multiple chronic conditions | Decem-ber 2012 | J Am Geriatric Society | USA | Agree om recommendations to address patient-centered health outcomes for older multiple chronic conditions (MCC) patients | Consensus meeting / Meeting | N/A | A panel of experts on health outcomes for older persons with MCC (n= 14) | The panel rerecommended several outcomes and how to measure them |
| Shankar *et al [15]* | Toward Patient-Centered Care: A Systematic Review of Older Adults’ Views of Quality Emergency Care | May 2014 | Annals of Emergen-cy Medicine | USA. | Synthesize the current knowledge about the elderly patient’s preferences and views of their emergency care. | Systematic review/ Review of qualitative studies and surveys/ Literature search in databases/ Meta-ethnography | Emergency care | Included articles with patients aged $\geq$65 years (n= 28) | Current qualitative research on the views of the elderly patient to hospital-based emergency care reveals common themes that should be considered in efforts to improve delivery of care to the elderly patient. |

| Stock *et al [16]* | Developing a Senior Healthcare Practice Using the Chronic Care Model. Effect on Physical Function and Health related Quality of life | July  2008 | J Am Geriatric Soc | USA | Investigate whether an interdisciplinary geriatric practice team in a primary care setting designed using the Chronic Care Model (CCM) produces better health status outcomes for Medicare beneficiaries | Observational longitudinal panel study/ Community-dwelling patients of clinics representing three different care models were followed across four data collection waves/ Telephone survey/ Mplus growth model approach | Community | Community-dwelling Medicare beneficiaries aged $\geq$66 years, with some level of physician care within the 12 months before baseline (n=1 709 (1307 completed the study)) | Despite physical function decline, patients in a senior health clinical care model, maintained health-related quality of life over time, whereas patients receiving traditional care had physical function and health-related quality of life decline |
| --- | --- | --- | --- | --- | --- | --- | --- | --- | --- |
| You *et al [17].* | Effects of case management in community aged care on client and carer outcomes: a systematic  review of randomized trials and comparative observational studies | 2012 | BMC Health Services Research | Australia | Evaluate the effects of case management in community aged care on client and carer outcomes | Systematic review/ Literature search in databases/Narrative summary of findings | Community | Randomized control trials and comparative observational studies with focus on community-dwelling frail older people were included (n= 15) | Available evidence showed that case management in community aged care can improve client  psychological health or well-being and unmet service needs. Future studies should investigate what specific components of case management are crucial in improving clients and their carers’ outcomes |

| Wald *et al [18]* | A patient-centered research agenda for the care of the acutely ill older patient | May  2015 | J Hosp Med | USA | Develop a research agenda to bridge the gap in evidence addressing the needs of the acutely ill older adult | Collaborative and consultative approach to stakeholder engagement/  Convene and consult of stakeholders, prioritize research questions/ Survey, meeting | Emergency medicine | Stakeholder organizations representing, patients, caregivers, advocacy organizations etc.  (n= 18) | A prioritized research agenda with the top 10 rated research questions was developed |
| --- | --- | --- | --- | --- | --- | --- | --- | --- | --- |

1. Akpan A, Roberts C, Bandeen-Roche K, Batty B, Bausewein C, Bell D, Bramley D, Bynum J, Cameron ID, Chen L-K *et al*: **Standard set of health outcome measures for older persons**. *BMC Geriatr* 2018, **18**(1):36.

**References**

2. Bayliss EA, Ellis JL, Shoup JA, McQuillan DB, Steiner JF, Zeng C: **Association between Continuity of Care and Health-Related Quality of Life**. *J Am Board Fam Med* 2017, **30**(2):205-212.

3. Bayliss EA, Ellis JL, Shoup JA, Zeng C, McQuillan DB, Steiner JF: **Association of Patient-Centered Outcomes With Patient-Reported and ICD-9-Based Morbidity Measures**. *Ann Fam Med* 2012, **10**(2):126-133.

4. Bearon LB, Crowley GM, Chandler J, Robbins MS, Studenski S: **Personal Functional Goals: A New Approach to Assessing Patient-Centered Outcomes**. *J Appl Gerontol* 2000, **19**(3):326-344.

5. Berg AI, Hoffman L, Hassing LB, McClearn GE, Johansson B: **What matters, and what matters most, for change in life satisfaction in the oldest-old? A study over 6 years among individuals 80+**. *Aging Ment Health* 2009, **13**(2):191-201.

6. Drouin H, Walker J, McNeil H, Elliott J, Stolee P: **Measured outcomes of chronic care programs for older adults: a systematic review**. *BMC Geriatr* 2015, **15**(1):139.

7. Berglund H, Hasson H, Kjellgren K, Wilhelmson K: **Effects of a continuum of care intervention on frail older persons’ life satisfaction: a randomized controlled study**. *J Clin Nurs* 2015, **24**(7-8):1079-1090.

8. Hong SH, Liu J, Tak S, Vaidya V: **The impact of patient knowledge of patient-centered medication label content on quality of life among older adults**. *Res Social Adm Pharm* 2013, **9**(1):37-48.

9. Kane AR, Lum YT, Cutler JL, Degenholtz BH, Yu T: **Resident Outcomes in Small-House Nursing Homes: A Longitudinal Evaluation of the Initial Green House Program**. *J Am Geriatr Soc* 2007(55:832-839).

10. Lee W-J, Peng L-N, Lin C-H, Lin S-Z, Loh C-H, Kao S-L, Hung T-S, Chang C-Y, Huang C-F, Tang T-C *et al*: **First insights on value-based healthcare of elders using ICHOM older person standard set reporting**. *BMC Geriatr* 2020, **20**(1):335.

11. Lee H, Shi SM, Kim DH: **Home Time as a Patient-Centered Outcome in Administrative Claims Data: Home time as a patient-centred outcome**. *J Am Geriatr Soc* 2019, **67**(2):347-351.

12. Leff B, Sheehan OC, Harrison KL, Eaton England A, Mickler A, Basyal PS, Garrigues SK, Schuchman M, Perissinotto C, Garrett SB *et al*: **A Home-Based Care Research Agenda by and for Homebound Older Adults and Caregivers**. *J Appl Gerontol* 2021, **40**(12):1715-1721.

13. Roberts TJ, Gilmore-Bykovskyi A, Lor M, Liebzeit D, Crnich CJ, Saliba D: **Important Care and Activity Preferences in a Nationally Representative Sample of Nursing Home Residents**. *J Am Med Dir Assoc* 2018, **19**(1):25-32.

14. Working Group on Health Outcomes for Older Persons with Multiple Chronic C: **Universal Health Outcome Measures for Older Persons with Multiple Chronic Conditions**. *J Am Geriatr Soc* 2012, **60**(12):2333-2341.

15. Shankar KN, Bhatia BK, Schuur JD: **Toward Patient-Centered Care: A Systematic Review of Older Adults’ Views of Quality Emergency Care**. *Ann Emerg Med* 2014, **63**(5):529-550.e521.

16. Stock R, Mahoney ER, Reece D, Cesario L: **Developing a Senior Healthcare Practice Using the Chronic Care Model: Effect on Physical Function and Health-Related Quality of life**. *J Am Geriatr Soc* 2008(56:1342-1348).

17. You EC, Dunt D, Doyle C, Hsueh A: **Effects of case management in community aged care on client and carer outcomes: a systematic review of randomized trials and comparative observational studies**. *BMC Health Serv Res* 2012, **12**(1):395.

18. Wald HL, Leykum LK, Mattison MLP, Vasilevskis EE, Meltzer DO: **A patient-centered research agenda for the care of the acutely Ill older patient: Research Agenda for Older Patient Care**. *J Hosp Med* 2015, **10**(5):318-327.
